# A Reperfusion BOLD-MRI Tissue Perfusion Protocol Reliably Differentiate Patients with Peripheral Arterial Occlusive Disease from Healthy Controls

**DOI:** 10.3390/jcm10163643

**Published:** 2021-08-18

**Authors:** Kristina Törngren, Stefanie Eriksson, Jonathan Arvidsson, Mårten Falkenberg, Åse A. Johnsson, Carl Sjöberg, Kerstin Lagerstrand, Joakim Nordanstig

**Affiliations:** 1 Department of Molecular and Clinical Medicine, Institute of Medicine, The Sahlgrenska Academy, University of Gothenburg, 413 90 Gothenburg, Sweden; 2 Department of Medical Radiation Sciences, Institute of Clinical Sciences, The Sahlgrenska Academy, University of Gothenburg, 413 90 Gothenburg, Sweden; stefanie.eriksson@vgregion.se (S.E.); jonathan.arvidsson@vgregion.se (J.A.); kerstin.lagerstrand@vgregion.se (K.L.); 3 Department of Medical Physics and Biomedical Engineering, Sahlgrenska University Hospital, 405 30 Gothenburg, Sweden; 4 Department of Radiology, Sahlgrenska University Hospital, 405 30 Gothenburg, Sweden; marten.falkenberg@vgregion.se (M.F.); ase.johnsson@vgregion.se (Å.A.J.); 5 Department of Radiology, Institute of Clinical Sciences, The Sahlgrenska Academy, University of Gothenburg, 413 90 Gothenburg, Sweden; 6 Antaros Medical, 752 37 Uppsala, Sweden; carl.sjoberg@antarosmedical.com; 7 Department of Vascular Surgery, Sahlgrenska University Hospital, 413 45 Gothenburg, Sweden

**Keywords:** peripheral arterial occlusive disease, atherosclerosis, intermittent claudication, BOLD MRI, tissue perfusion

## Abstract

There is no established technique that directly quantifies lower limb tissue perfusion. Blood oxygenation level-dependent magnetic resonance imaging (BOLD-MRI) is an MRI technique that can determine skeletal muscle perfusion. BOLD-MRI relies on magnetic differences of oxygenated and deoxygenated hemoglobin, and regional changes in oxy/deoxyhemoglobin ratio can be recorded by T2* weighted MRI sequences. We aimed to test whether BOLD-MRI can differentiate lower limb tissue perfusion in peripheral arterial occlusive disease (PAOD) patients and healthy controls. Twenty-two PAOD patients and ten healthy elderly volunteers underwent lower limb BOLD-MRI. Reactive hyperemia was provoked by transient cuff compression and images of the gastrocnemius and soleus muscles were continuously acquired at rest, during ischemia and reperfusion. Key BOLD parameters were baseline T2* absolute value and time to T2* peak value after cuff deflation (TTP). Correlations between imaging parameters and ankle-brachial index (ABI) was investigated. The mean TTP was considerably prolonged in PAOD patients compared to healthy controls (m. gastrocnemius: 111 ± 46 versus 48 ± 22 s, *p* = 0.000253; m. soleus: 100 ± 42 versus 41 ± 30 s, *p* = 0.000216). Both gastrocnemius and soleus TTP values correlated strongly with ABI (−0.82 and −0.78, *p* < 0.01). BOLD-MRI during reactive hyperemia differentiated most PAOD patients from healthy controls. TTP was the most decisive parameter and strongly correlated with the ABI.

## 1. Introduction

Peripheral arterial occlusive disease (PAOD) is a large and increasing public health issue [1]. PAOD is mainly a clinical diagnosis based on symptoms, clinical findings and evidence of hemodynamic compromise, with decreased or falsely increased ankle-brachial index (ABI). Further characterization of the ischemic state, feasibility of revascularization and pre-procedural planning is mainly based on vascular imaging techniques, including duplex ultrasound, computed tomography angiography (CTA), magnetic resonance angiography (MRA) and digital subtraction angiography (DSA) [2]. While all these imaging techniques currently provide high resolution images of the arterial tree, there is still no established imaging technique that directly quantifies the crucial pathophysiological process in PAOD—the tissue perfusion and, in particular, the oxygenation of peripheral skeletal muscle. As all revascularization procedures for PAOD ultimately aim to improve ischemic tissue perfusion, an imaging tool that can characterize and quantify tissue ischemia in the lower limb would potentially be of value both in the diagnostic process and in treatment outcome evaluations in PAOD patients.

Magnetic resonance imaging (MRI) technology for PAOD applications have developed substantially during recent years, including functional imaging techniques [3,4]. Blood oxygenation level-dependent magnetic resonance imaging (BOLD-MRI) is a noninvasive functional MRI technique that allows for the quantification of skeletal muscle perfusion without the use of contrast agents. The BOLD-signal depends on differences in the magnetic properties of oxygenated and deoxygenated hemoglobin. Regional changes in the oxy/deoxyhemoglobin ratio can be recorded by T2*-weighted MRI sequences and quantified using T2*-mapping for objective comparisons between subjects. Deoxygenated hemoglobin is paramagnetic and increased concentration in deoxygenated blood leads to a reduction in the T2* signal [5,6], whereas oxygenated hemoglobin is diamagnetic which leads to an increased T2* signal. It is believed that the changes in ratio between oxy- and deoxyhemoglobin can be measured by the BOLD-MRI technique as T2* sequences [7]. In muscle tissue perfusion imaging, the changes in ratio between oxygenated and deoxygenated blood can be attenuated by a transient cuff compression instituted proximally to the investigated area. Within neuroimaging, BOLD-MRI protocols can successfully characterize task-induced or spontaneous changes of brain metabolism in metabolically active brain areas [8]. Among healthy volunteers, preliminary BOLD-MRI imaging protocols that characterize foot muscle oxygenation have also been demonstrated to enable repeatable volumetric assessment of regional skeletal muscle foot perfusion [9]. A small feasibility study also previously showed that BOLD-MRI T2* time course signals during reactive hyperemia were different in PAOD patients compared with controls [10].

By definition, only the most severe form of PAOD (chronic limb-threatening ischemia, CLTI) exhibits a tissue perfusion defect at rest that causes symptoms (rest pain and/or tissue loss), whereas the tissue perfusion defect in patients with less severe ischemia (intermittent claudication (IC)) typically only causes symptoms during and immediately after exercise [11]. To mimic the clinical situation for PAOD patients, and to enhance the responsiveness of the test, BOLD-MRI protocols need to be complemented with a method that induces reactive hyperemia [12]. A compression with a blood pressure cuff at the level of the thigh that temporarily interrupts lower limb arterial blood flow can induce ischemia reperfusion during the BOLD-MRI examination. The temporarily interrupted blood flow followed by reperfusion can be measured with the BOLD-MRI technique, allowing the quantification of the arterial perfusion of the muscle [10,13]. While it is conceivable that several of these dynamic signal responses might hold key insights into the physiological state of the tissue microvasculature response, this study specifically focused on the reperfusion phase, and explored the hypothesis that the quantitative BOLD-MRI signal changes during reperfusion would reliably differentiate lower extremity tissue perfusion between patients with PAOD and healthy controls.

## 2. Materials and Methods

### 2.1. Study Participants

A representative sample of twenty-two patients (mean age 73.5 ± SD 4 y; 12 male) with confirmed PAOD (Rutherford stage 1–3; symptoms > 6 months) and ten healthy elderly volunteers (mean age 67.9 ± SD 6.2 y; 8 male) with normal ABI and no IC symptoms were included in the study. All individuals underwent a clinical evaluation including ABI measurements at rest, followed by a BOLD-MRI-examination. All participants in the study provided written informed consent and the study protocol was approved by the Regional Ethical Review Board in Gothenburg (entry no. 1157-17). 

### 2.2. BOLD-MRI Examinations

The BOLD-MRI examination was performed on a clinical 3 T whole-body scanner (Magnetom Skyra, Siemens Healthineers, Erlangen, Germany) with a large 4-channel flex coil. 

**Figure 1:**
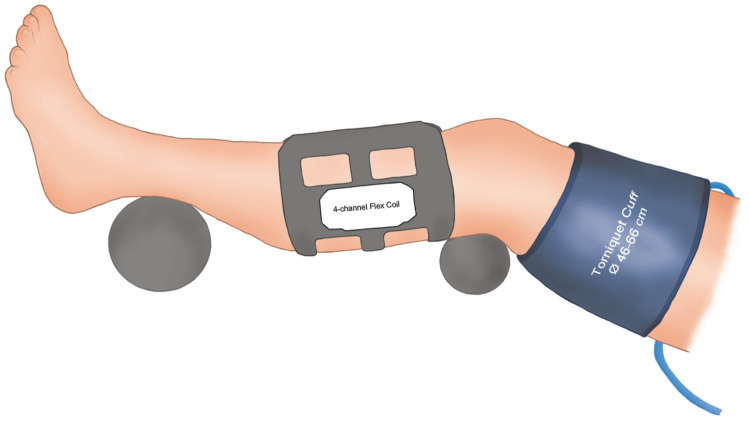
Schematic illustration of the positioning of the lower limb during the BOLD-MRI examination.

Each participant was examined in a supine position with their most symptomatic lower leg supported both at the knee and foot level to reduce any external loading on the calf. A receiver flex coil was thereafter wrapped around the calf. To reduce calf muscle deformation, a thin piece of foam rubber was placed between the coil and the calf (Figure 1).

The current brachial blood pressure was measured at the day of MRI scanning. All participants were at rest for at least 10 min before scanning. MRI measurements were performed for 660 s using a 2D multi-echo gradient echo, see the scan protocol in Table 1. The images were collected from the widest part of the calf.

Reperfusion during the BOLD-MRI diagnostic procedure was provoked by transient compression using an inflatable thigh cuff (ERKA, Berlin, Germany) and an automatic tourniquet insufflation device (Zimmer MerdizinSystems, ATS 750, Irvine, CA, USA) that was placed outside of the MRI room. The cuff was fixed at mid-thigh. Rapid inflation was done one minute after the T2*-measurements started. Inflation pressure was pre-set to 50 mmHg above the recorded systolic pressure of the right arm. After five minutes, the tourniquet pressure was rapidly released. The T2*-images were continuously acquired with a temporal resolution of 3.2 s at rest (1 min), during compression (5 min) and during the reperfusion phase (5 min).

### 2.3. Image Post Processing 

**Figure 2:**
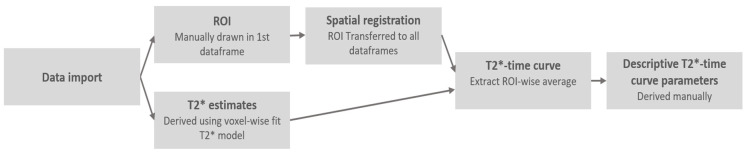
Schematic of image post processing software, where T2* denotes the effective T2 relaxation time. ROI denotes regions of interest.

The post-processing of all images was performed using a dedicated analysis software for calf muscle perfusion written in Python (Python Software Foundation. Python Language Reference, version 3.7.5. Available at http://www.python.org, accessed on 3 May 2020), Cython [14] and Jupyter Notebooks [15]. Following the schematic in Figure 2, the data import was followed by the derivation of dynamic T2*-maps from the single-slice measurement. The T2*-estimates were estimated pixel-wise using a weighted least square fitting to a negative exponential signal decay model. 

**Figure 3:**
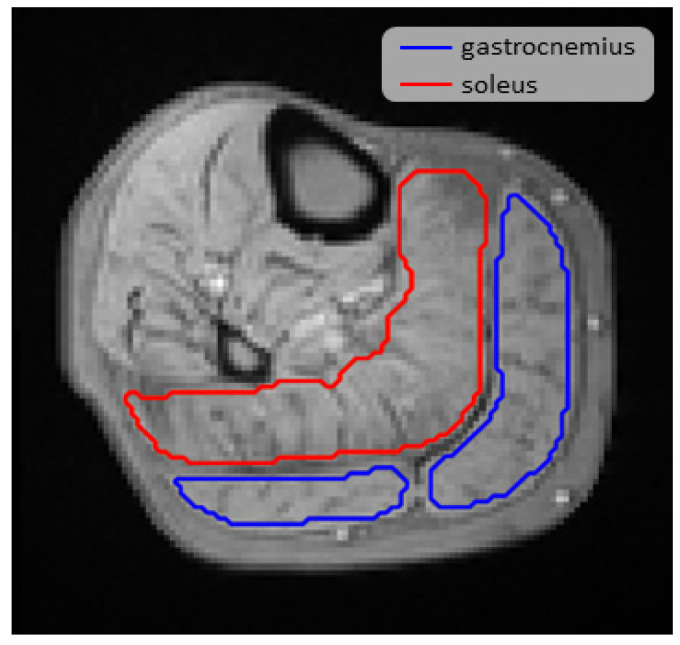
Regions of interest (ROIs) drawn on a T2*-weighted image. The blue ROIs are drawn in the gastrocnemius muscle (lateral and medial head) and the red ROI is drawn in the soleus muscle.

The perfusion T2*-time course in the gastrocnemius and the soleus muscles were determined, using manually outlined regions of interest (ROIs) in the dynamic T2*-maps, including 700 to 1600 pixels. The ROIs were hand-drawn using SmartPaint [16], keeping a safety margin to the border of the muscle groups (Figure 3).

**Figure 4:**
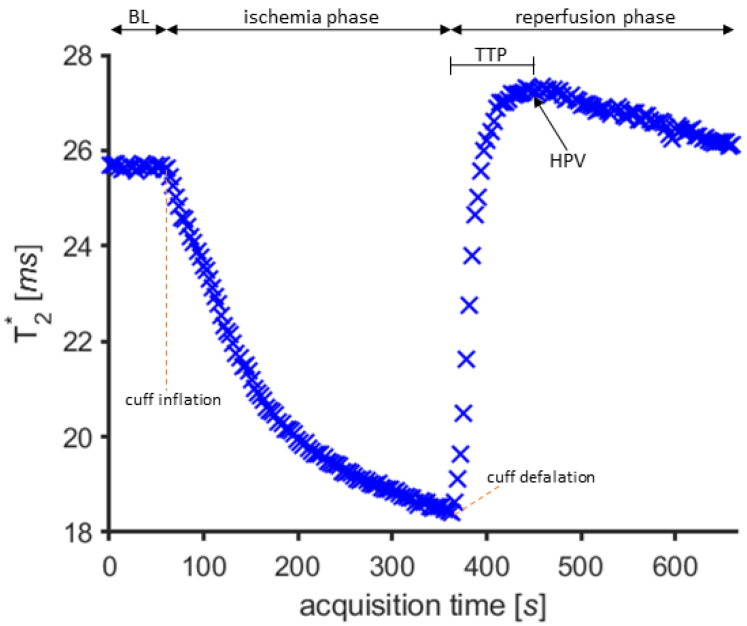
T2*-time curve of the soleus muscle in a PAOD patient, showing the dynamic response during the three phases of the examination (baseline, ischemia and reperfusion), and corresponding descriptive measures at baseline/resting phase during 60 s (BL), hyperemia peak absolute value (HPV) and reperfusion time to peak (TTP).

A spatiotemporal registration was performed to correct for subject motion during scanning. This was carried out by registering the first echo time frame to the corresponding image of each consecutive acquisition and applying the resulting transformations to the ROIs accordingly. Registrations were performed using Elastix [17] and ITK-snap [18]. Finally, ROI-mean T2*-time curves were extracted and plotted to display the level of tissue oxygenation throughout the cuffing paradigm. From these T2*-time curves, a number of descriptive curve features were identified. For this study, the T2*-value during baseline, characterizing the resting state of the muscle, the T2*-peak value after deflation (hyperemia peak value), and the time to peak (TTP) of the BOLD signal during reperfusion were the primary variables of interest (Figure 4).

### 2.4. Statistical Analysis

Descriptive statistics are presented as absolute numbers and relative frequencies. Non-parametric testing (Mann Whitney U test) was used to compare the different BOLD-MRI parameters between patients and healthy controls. Main results are displayed in box plots and significance was assumed at *p* < 0.05. Correlations between the imaging parameters of interest and the ABI were assessed using the Spearman rank correlation test, where a value of 0.10–0.39 was considered to be weak; 0.40–0.69 moderate; 0.70–0.89 strong; 0.90–1.00 very strong [19]. Statistical analysis was performed using SPSS^®^ version 27 (IBM, Armonk, NY, USA).

## 3. Results

### 3.1. Study Population

Baseline characteristics for all study participants are displayed in Table 2. As expected, patients with PAOD also had a more pronounced cardiovascular risk profile. Among the PAOD patients, the majority had moderate IC symptoms (Rutherford stage 2) and the maximal walking distance on a graded treadmill test was mean (SD) 167 (64) meters.

### 3.2. BOLD-MRI Examination Results 

**Figure 5:**
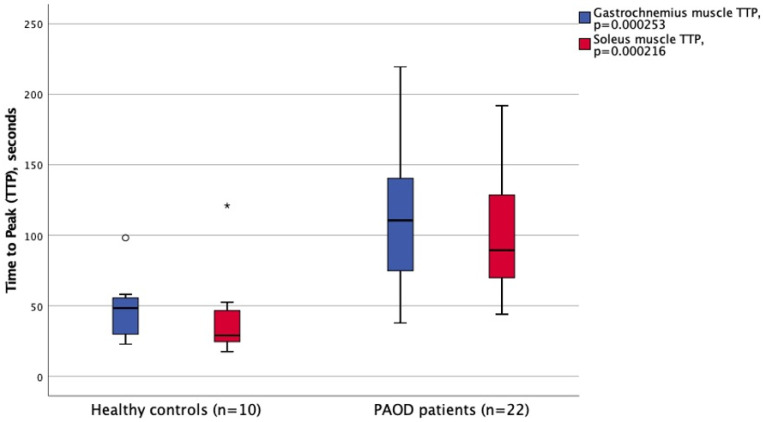
Comparison of the time to peak (TTP) parameter between healthy controls and PAOD patients in the gastrocnemius (blue) and the soleus (red) muscles. Box and whiskers plots revealed significantly prolonged TTP in PAOD patients as compared to healthy controls (*p* < 0.001); and a high discrimination capacity (PAOD patients versus controls) by the BOLD-MRI protocol. The whiskers indicate observed minimum and maximum values. * and ^o^ denotes outliers.

All MRI examinations were performed successfully, and the compression was well tolerated by all study participants. The time to peak (TTP) value after deflation was considerably prolonged in PAOD patients as compared to healthy controls, both in the gastrocnemius and soleus muscles (m. gastrocnemius: 111 ± 46 versus 48 ± 22 s, *p* = 0.000253; m. soleus: 100 ± 42 versus 41 ± 30 s, *p* = 0.000216), Figure 5. No difference between patients and controls was observed in neither the absolute baseline T2*parameter nor the absolute hyperemia peak value. However, the observed changes from T2* baseline value to hyperemia peak value (HPV minus BL = overshoot) were lower in PAOD patients than in healthy controls, but this was only significant in the gastrocnemius muscle. T2* hyperemia peak value minus baseline T2* value was 0.59 ± SD 0.93 in PAOD patients and 1.49 ± 1.22 ms in healthy controls (Table 3).

### 3.3. Correlations between Reperfusion BOLD-MRI Parameters and ABI

Strong correlations were observed between both the gastrocnemius and soleus TTP values and the ankle-brachial index (−0.78 and −0.75, *p* < 0.01).

## 4. Discussion

In this study, we evaluated the feasibility of using a reperfusion BOLD-MRI protocol to distinguish between patients with confirmed PAOD and healthy controls. Most patients were clearly differentiated from healthy controls using this BOLD-MRI protocol. The most discriminative parameter analyzed in this study was the TTP value during reactive hyperemia. This highly discriminative parameter, as measured both in the gastrocnemius and soleus muscles, was also strongly correlated with the ankle-brachial index. We also observed numerically lower overshoot values among PAOD patients in both evaluated muscles groups, but this reached statistical significance only in the gastrocnemius muscle. These preliminary results therefore lend some support to the theory that functional MRI techniques may add value in the overall diagnosis and management of PAOD patients. 

Previous studies have demonstrated differential BOLD-MRI perfusion parameter responses in different muscles groups in patients both with and without PAOD [20,21]. This variation may be attributed to differences in physical activity, smoking status and/or age-related structural and metabolic changes in skeletal muscles [22,23,24]. Possible confounding effects that can influence T2* signal changes in muscle tissue, such as age, smoking state, muscle fiber types and simultaneous diabetes or renal disease, are of interest to study further. It is believed that tissue oxygenation state is multifactorial and can be influenced by lactate, blood volume or hemoglobin concentration. Muscle flow response was studied by Nishii et al. using the BOLD-MRI technique in young smokers and non-smokers and significantly higher TTP values were observed in non-smokers [24]. That could potentially be explained by the pathophysiological changes in muscle tissue induced by smoking (i.e., increased oxygen extraction) rather than structural tissue changes in this young population. We would assume that the significantly prolonged TTP in patients with PAOD that was observed in our study is the result of adaptations in both muscle metabolism and structural re-arrangements in muscle tissue as a result of the chronic ischemic state induced by PAOD. Currently, these intriguing pathophysiologic responses are still not fully understood. Furthermore, recent research by Guensch et al. has shown that myoglobin (Mb) desaturation may also play into the measured signal, although Mb desaturates later than Hb [25]. However, even a compound measure of Hb and Mb desaturation may be of importance in the characterization of PAOD as suggested by our results. 

Although it might be argued that other and less complicated diagnostic tools as compared to BOLD-MRI can also differentiate PAOD patients from patients without PAOD, there are several situations in clinical reality where an objective assessment of the lower limb ischemic situation with conventional methods (such as the ABI, toe pressures or transcutaneous pO2 measurements) remains problematic. This includes the clinical diagnosis of patients with stiff, incompressible infrapopliteal arteries due to medial arterial layer calcification (commonplace in diabetes and chronic kidney disease), as well as when evaluating revascularization results in patients with significant multi-level lower limb arterial disease and where only a proximal revascularization procedure has been undertaken (for instance an aorto-iliacal intervention to improve profundal artery collateral flow in a patient with significant remaining superficial and/or popliteal artery atherosclerosis). Another area where BOLD-MRI tissue perfusion protocols might, at least in theory, add clinical value is in patients with other concomitant diagnoses that may also lead to significant exercise induced lower limb symptoms (osteoarthritis, spinal stenosis etc.) alongside the presence of PAOD as determined by a reduced ABI. In such clinical situations, the actual root cause of the lower limb symptoms might be very challenging to determine, which is why a direct and quantifiable measurement method of tissue perfusion may ultimately provide better guidance for subsequent therapy choices. Finally, BOLD-MRI protocols may add value to assess peripheral circulation in certain patients at high risk for amputation that, due to intercurrent disease, do not exhibit IC or rest pain symptoms (i.e., heart failure patients or patients with peripheral neuropathy). In such populations, these sophisticated MRI techniques may be important for identifying a perfusion defect at an earlier stage, which may provide a treatment window that might in theory modify subsequent limb outcomes. BOLD-MRI sequences may also be a valuable addition to pathoanatomical evaluations of lower limb arteries by established MR angiography protocols, as it provides quantifiable physiologic information about deep tissue perfusion in the leg. The BOLD MR technique has been studied quite scarcely in PAOD. In a similar set-up as in our study, Potthast et al. demonstrated that a significantly reduced BOLD signal decreases during compression compared to a control group, and attributed this observation to pathological changes in muscle structure and/or metabolism in PAOD patients [20]. Other functional MRI techniques have also been suggested in PAOD applications, including arterial spin labeling techniques [4]. Furthermore, CT perfusion techniques are under development and may provide reasonable alternatives to quantify PAOD tissue perfusion [26,27,28]. To the best of our knowledge, no functional MRI technique has been broadly introduced in clinical practice or included in pathoanatomical PAOD imaging protocols, perhaps due to logistical and technical issues including limited availability of MRI scanners, problems with signal strengths in smaller muscles, risk for motion artifacts and the added time for image acquisition. The herein suggested BOLD-MRI protocol is quite easy to set up, and was overall well tolerated by patients, so other clinicians and researchers should be able to replicate and further develop these techniques. However, it should be recognized that BOLD-MRI techniques and other functional MRI techniques are still under development for PAOD applications and the proper place for these advanced imaging techniques in PAOD management algorithms remains to be established [4,9,11]. Important areas for future studies are to test how BOLD-MRI may be implemented in CLTI, the most severe form of PAOD, and whether resting T2* signal values could be used to quantify the perfusion deficit that, by definition, is present at rest in these patients (i.e., avoiding the need for thigh tourniquet compression during the MRI examination). The potential for BOLD-MRI imaging to predict wound healing in CLTI remains to be studied, but also here, BOLD-MRI and other functional MRI techniques may, in the near future, guide indications for lower limb revascularization by establishing new thresholds where wound healing will not be achievable without an improvement in tissue perfusion [29]. Further, BOLD-MRI may potentially be a useful tool to evaluate the lower limb collateral capacity following proximal vascular interventions when significant atherosclerotic disease remains lower down in the arterial tree. Finally, TPP measurements of the gastrocnemius and soleus perfusion produced the same statistically significant result, which may mean that the method can be simplified by analyzing only one muscle group, which is easiest to delimit in the image.

Limitations in this study include the somewhat small sample size; and it has to be recognized that this, by design, was a “proof of concept” study. Still, within this limited sample, our reperfusion BOLD-MRI protocol was able to discriminate PAOD patients from healthy controls. Furthermore, a substantial proportion of PAOD patients in our study had concurrent diabetes, and although this is quite representative of a contemporary revascularized PAOD cohort, we cannot exclude that the BOLD signal patterns might be different in PAOD patients with media sclerosis as compared to PAOD patients without such pathology. To study BOLD signal pattern differences in PAOD patients with and without diabetes represents an important research objective for future studies. Another limitation is that we did not study how the BOLD-MRI parameters during reperfusion correlated with the outcomes most important to PAOD-patients, such as walking capacity and health-related quality of life, but we will investigate this in future studies. Moreover, we only studied PAOD patients with clinically moderate disease (i.e., intermittent claudication) and our findings may therefore not be extrapolated to the more severely diseased CLTI population.

## 5. Conclusions

The reperfusion BOLD-MRI protocol differentiated most patients with PAOD from healthy controls. Further studies are warranted to investigate whether BOLD-MRI may add value in clinical routine scenarios, such as when determining indications for revascularization and as a technique to evaluate changes in tissue perfusion after therapeutic procedures.

## Figures and Tables

**Table 1:** Protocol parameters for the BOLD-MRI examination.

Parameter	
Field-of-view (mm)	160 × 160
Acquisition matrix	128 × 119
TR (ms)	44
TE_1–11_ (ms)	2–40
Slice Thickness (mm)	10

TR = repetition time; TE_1–11_ = echo times for echoes 1 to 11 (2, 5.8, 9.6, 13.4, 17.2, 21, 24.8, 28.6, 32.4, 36.2, 40).

**Table 2:** Baseline characteristics for study participants.

	Patients with PAOD (*n* = 22)	Controls without PAOD (*n* = 10)
Male sex	12 (55%)	8 (80%)
Smoking:		
active smoker	2 (9%)	1 (10%)
previous smoker	17 (77%)	4 (40%)
never smoked	3 (14%)	5 (50%)
Hypertension	18 (82%)	3 (30%)
Hyperlipidemia	11 (50%)	2 (20%)
Diabetes	9 (41%)	0 (0%)
Coronary artery disease	11 (50%)	0 (0%)
Chronic obstructive pulmonary disease	6 (27%)	0 (0%)
Venous insufficiency	1 (5%)	3 (30%)
Rutherford stage:		
Stage 1	5 (23%)	N/A
Stage 2	14 (64%)	N/A
Stage 3	3 (14%)	N/A
Ankle-brachial index at rest, mean (SD)	0.65 (0.23)	1.13 (0.09)

N/A = not applicable, PAOD = peripheral arterial occlusive disease, SD = standard deviation.

**Table 3:** Mean T2* perfusion values in the gastrocnemius and the soleus muscles, presented separately for patients with PAOD and control group.

BOLD-MRI Perfusion Parameter	Patients with PAOD (*n* = 22)	Controls without PAOD (*n* = 10)	*p*-Value
BL (ms) gastrocnemius	25.3 ± 2.9	24.6 ± 4.1	0.59
BL (ms) soleus	21.2 ± 2.8	20.8 ± 3.9	0.91
HPV (ms) gastrocnemius	25.8 ± 2.5	26.1 ± 4.3	0.31
HPV (ms) soleus	22.8 ± 2.7	22.6 ± 4.3	0.96
TTP (s) gastrocnemius	111 ± 46	48 ± 22	<0.01
TTP (s) soleus	100 ± 42	41 ± 30	<0.01
Overshoot (ms) gastrocnemius	0.59 ± 0.93	1.49 ± 1.22	0.03
Overshoot (ms) soleus	1.59 ± 0.96	1.81 ± 1.86	0.46

Overshoot = T2* hyperemia peak absolute value minus baseline T2* absolute value; BL = baseline, HPV = hyperemia peak value, TTP = time to peak value. Values represent mean values ± standard deviation (SD).

## Data Availability

The data presented in this study are available on request from the corresponding author. The data are not publicly available due to ethical restrictions.
